# Intra‐articular and intra‐osseous expanded adipose‐derived stromal cell injections for knee osteoarthritis‐related bone marrow lesions yield promising outcomes: Preliminary results in 16 athletes

**DOI:** 10.1002/jeo2.70527

**Published:** 2026-01-14

**Authors:** Miguel A. Khoury, Emmanuel T. Papakostas, Theodorakys Marín Fermín, Montassar Tabben, Karim Chamari, Lorena Levi, Khaled Alkhelaifi, Pieter D'Hooghe

**Affiliations:** ^1^ Surgery Department Aspetar Orthopaedic and Sports Medicine Hospital Doha Qatar; ^2^ Orthopedic Department Clínica Santa Sofía, Av. Principal de Santa Sofía El Cafetal Caracas Venezuela; ^3^ Research Department, Olympic Medical and Scientific Committee Qatar Olympic Committee Doha Qatar; ^4^ Research Department, Naufar Wellness and Recovery Center Doha Qatar; ^5^ Research Department, Higher Institute of Sport and Physical Education, ISSEP Ksar Saïd Manouba University Tunis Tunisia

**Keywords:** Athlete's knee injections, bone marrow lesions, degenerative joint disease, intra‐osseous injection, mesenchymal stromal cells

## Abstract

**Purpose:**

To explore the results of a fluoroscopic‐guided intra‐osseous (IO) and intra‐articular (IA) injection of expanded adipose‐derived stromal cells (ASCs) in athletes with bone marrow lesions (BML) associated with knee osteoarthritis (OA).

**Methods:**

A prospective study was conducted on 16 athletes (9 males, 7 females; mean age 30.5 ± 2.8 years), including two with bilateral knee involvement. All had Kellgren‐Lawrence grades 2–4 and received IA and IO injections of 24.3 ± 2.1 million and 23.4 ± 1.9 million ASCs, respectively. Clinical outcomes were assessed using the Knee Injury and Osteoarthritis Outcome Score (KOOS), Numeric Pain Rating Scale (NPRS), and Tegner activity scale at baseline, 6 months, and 12 months. Radiological changes were evaluated using Magnetic Resonance Imaging Osteoarthritis Knee Scores (MOAKS) at baseline and 12 months.

**Results:**

Significant improvements were observed in NPRS, Tegner, and all KOOS subscales at 6 months and 12 months with respect to baseline (*p* < 0.05). At 12 months, NPRS decreased from 6.6 ± 0.9 to 2.4 ± 0.5, and Tegner increased from 4.4 ± 1.8 to 7.8 ± 1.1. All KOOS subscale scores improved at 12 months. Symptoms: 49.3 ± 6.4 to 74.1 ± 4.9; pain: 49.4 ± 4.3 to 75.8 ± 3.2; sport and recreation: 30.6 ± 6.2 to 67.8 ± 4.3; activities of daily living: 57.6 ± 3.6 to 79.9 ± 4.8; and quality of life: 35.4 ± 10.3 to 66.3 ± 5.3. A mild decline yet significant in the KOOS sport and recreation subscale was noted between 6 and 12 months (*p* = 0.02), but remained significantly improved from baseline. MOAKS analysis showed improvement in 12 of 27 evaluated regions. No major complications occurred.

**Conclusions:**

Combined IA and IO ASC injections are a safe and effective treatment for OA‐BMLs in athletes, offering sustained clinical and radiological benefits over 12 months.

**Level of Evidence:**

Level III.

**Abbreviations**
ASCs: adipose‐derived mesenchymal stromal cells
BMAC: bone marrow aspirate concentrate
BME: bone marrow oedema
BMLs: bone marrow lesions
HBSS: Hanks balanced salt solution
IA: intra‐articular
IO: intra‐osseous
K‐L: Kellgren‐Lawrence
KOOS: Knee Injury and Osteoarthritis Outcome Score
MCID: minimal clinically important difference
MOAKS: Magnetic Resonance Imaging Osteoarthritis Knee Score
MRI: magnetic resonance imaging
MSCs: mesenchymal stromal cells
NPRS: Numeric Pain Rating Scale
OA: osteoarthritis
PRP: platelet‐rich plasma
WORMS: Whole‐Organ Magnetic Resonance Imaging Score

## INTRODUCTION

Bone marrow oedema (BME) is a subchondral alteration detected by magnetic resonance imaging (MRI) that is invisible to radiographs and ultrasound. They are characterised by a high signal intensity on fluid‐sensitive sequences, such as T2‐weighted, fat‐suppressed, and short tau inversion recovery (STIR) images [9, 35, 47]. BMEs are nonspecific and can result from trauma, overuse injuries in athletes, infection, or neoplasia [43]. One of the most common varieties is that associated with osteoarthritis (OA) [46], which typically indicates deeper, subchondral bone pathologic alterations such as fibrosis, marrow necrosis, abnormal trabecular structure, trabecular thickening or remodelling, and increased vascularity, presenting with pain and functional impairment, and collectively termed bone marrow lesions (BMLs) [3, 11, 20, 39].

Given the correlation between OA‐BMLs and knee joint pain and degeneration, its presence in athletes represents a clinical challenge. Pain and disability reduce athletic performance and impact the athletes' quality of life. Furthermore, concerned athletes face an increased risk of disease progression and eventual need for surgical interventions, including knee arthroplasty [26, 42]. Current treatment options include intraosseous injections of platelet‐rich plasma (PRP) [7, 23, 27, 37, 38, 41, 45] or bone marrow aspirate concentrate (BMAC) [5, 19, 22], which have shown satisfactory results. However, evidence on the treatment with adipose‐derived stromal cell (ASCs) intraosseous (IO) injections remains scarce.

ASCs possess several biological capacities, including the expression of immunomodulatory cytokines, the regulation of chondrocytes, and the activation of protective mechanisms [29, 31]. They can be easily obtained from adipose tissue and, after expansion, represent a potential therapy for treating BMLs, even when injected intra‐articularly [16, 36]. Combining intra‐articular (IA) and IO ASCs injections may offer synergistic benefits by targeting both cartilage and subchondral bone. Therefore, this study aimed to assess the clinical and radiologic outcomes of fluoroscopy‐guided IA and IO injections of ASCs in athletes with knee OA‐BMLs. We hypothesise that this therapeutic approach would reduce symptoms, improve function, and alter the disease progression in this population.

## METHODS

This prospective case series study was approved by the Ethical Committee of Regenerar® (Clinical Trial MR0024).

### Participants

Consecutive participants (young active and professional athletes older than 18 years old) with knee pain and confirmed OA‐BMLs were referred to our institution from January 2021 to February 2023. Patients were considered for IA and IO injections of ASCs after being assessed by the senior author, and all participants signed informed consent to participate.

### Inclusion and exclusion criteria

Patients were considered eligible for the study if they had knee OA Kellgren‐Lawrence (K‐L) grade I–IV; less than 5° varus or valgus knee deformity as measured by the long mechanical axis of the knee on X‐ray; failed conservative treatment after 3 months of a protocol that included medications (analgesics, anti‐inflammatory drugs and/or supplements), counselling (individualised exercise programmes and nutritional advice), and/or orthotics when necessary; a minimum Numerical Pain Rating Scale (NPRS) score of 5 (out of 11 points); the presence of an increased signal intensity lesion (>1 cm in diameter) in T2‐weighted fat suppression images located in the subchondral region of the proximal tibia, patella or femoral condyles; and they were able to understand and complete the patient‐reported outcome questionnaires.

Exclusion criteria were: having knee pain from causes different than OA (i.e., tumours or referred pain from the hip or lumbar spine); previous surgery within the last 12 months before enroling in the study; previous mesenchymal stromal cell (MSCs) therapy due to any medical condition; a displaced meniscal tear, or meniscal extrusion on MRI; previous knee IA injectable therapy within the last 6 months; coagulation disorders (i.e., haemophilia); a history of cancer, systemic illness or significant organ impairment/failure (i.e., renal failure) and/or an atypical chronic pain syndrome (i.e., chronic regional pain); history of allergy to any substances used within the treatments; history of acute trauma and BME in the past 12 months); and/or pregnant or breastfeeding females.

### Data collection and outcome measures

Baseline patient demographics and prior surgical treatment were recorded. Additionally, the lower extremity mechanical axis was measured using standing panoramic views in a standard fashion [25], and knee OA using the K‐L grading system was evaluated with an X‐ray [18].

#### Clinical scores

Knee Injury and Osteoarthritis Outcome Score (KOOS) [24], NPRS [10], and Tegner scale [44] were prospectively evaluated, in the patients' native language, with validated translations, at baseline, 6‐ and 12‐month posttreatment.

Minimal Clinically Important Difference (MCID), defined as the slightest difference between two measurements deemed necessary for a patient to perceive beneficial clinical improvement, was calculated for KOOS subscales as the percentage of patients that perceive clinical improvement with differences between 6‐month posttreatment respect pretreatment greater than 9.3 for pain improvement, 8.4 for symptoms, 9 for activities of daily living (ADL), 12.5 for sport and recreation, and 10.3 for quality of life (QoL); and between 12‐month posttreatment respect pretreatment greater than 9.1 for pain improvement, 8.2 for symptoms, 9.2 for ADL, 11.6 for sport and recreation, and 10.3 for QoL [4, 15].

#### MRI

An experienced Musculoskeletal radiologist assessed the BMEs and cartilage status using the MRI Osteoarthritis Knee Score (MOAKS) at baseline and at the 12‐month follow‐up. MOAKS is a standardised, reproducible, validated, semi‐quantitative scoring system for knee OA evaluation. MOAKS includes 14 knee subregions and scores bone marrow and articular cartilage from 0 to 3 grades [12, 40]. The equipment used was a 3.0‐T system with a dStream MRI 5300 scanner Flex‐M Coil (Philips). Short tau inversion recovery (STIR) coronal, T1‐weighted, and T2‐weighted fat‐suppressed images in axial and sagittal views (field of view [FOV] = 130 mm, TR = 3500 ms, TE = 50 ms, TI = 100 ms) were evaluated, with a slice thickness of 3 mm.

### Intervention

#### Adipose‐derived stromal cell harvesting, isolation and expansion

Before tissue collection, the patients were examined for their normal prothrombin time, partial thromboplastin time and platelet count. Adipose tissue was obtained from the periumbilical zone. The procedure was done in an outpatient setting using local anaesthesia (lidocaine 2% with epinephrine). A total of 2–5 mL of adipose tissue was obtained by multiple punctures with BARD MAGNUM™ Reusable Core Biopsy Instrument (BD®) and the corresponding biopsy needles.

Samples were collected in sterile Hanks Balanced Salt Solution (HBSS) (Cat. #14,025,088; Gibco®). Adipose tissue for isolation and culture was rinsed with HBSS and incubated with Neutral Protease NB GMP Grade (Cat. #DS30303.01, SERVA®). MSCs were counted and seeded in a T25 25 cm^2^ tissue culture flask in complete media supplemented with 100 IU penicillin, 100 IU amphotericin, and 100 IU streptomycin (Ritcher), 10% autologous serum. When cultures reached 90%–100% confluence, subculturing was performed using trypsin TrypLE Select (Cat. #12,563,029; Gibco®). When detached, the cells were at passages of 3 or lower.

Cell count and viability were confirmed using a manual hemocytometer. In the laboratory facility, ASCs were successfully cultured. These cells exhibit elongated nuclei and spindle‐shaped cytoplasm, characteristic of a fibroblast‐like morphology. To determine surface marker expression, ASCs were characterised with antibodies against CD73, CD90, CD105, CD45, CD34, CD11b, CD19 or CD79a, CD14 and HLA class II (6) using the FACSCanto™ II (BD) flow cytometer. The samples were immunostained with the aforementioned monoclonal antibodies conjugated with different fluorochromes, and mouse isotype antibodies were used as controls. The samples used were from athletes who were to be injected. The results were analysed using the software Infinicyt® version 1.7i.

#### Adipose‐derived stromal cell intraosseous and intra‐articular injection

Prior to injection, ASCs were tested for endotoxin, mycoplasma and microbial contamination. ASCs were then washed twice and suspended in HBSS (For injection, MSCs were washed and suspended in 5 mL HBSS without antibiotics) [16].

The injections were performed by the senior author, under sedation and sterile conditions on a radiolucent table with the patient in a supine position, without a tourniquet. IA injections were performed via a superolateral approach and IO targeting the BMEs in the femur, tibia, and/or patella, based on the initial MRI assessment. The operative knee was positioned to facilitate intraoperative fluoroscopic imaging [34]. General sedation was used. Local anaesthetic (lidocaine 2%) was injected in the skin, and a mean of 47.7 ± 0.2 million ASCs were injected in each affected knee (24.3 million IA and 23.4 million IO). Cell count was confirmed using the Scepter™ instrument (MSD®) immediately prior to the injection.

#### Postprocedure protocol

Immediately after the procedure, the athletes were cleared for full weight‐bearing using crutches for ~24 h, if needed. Physical therapy was initiated one day after the procedure. Passive and active mobilization were started early in the rehabilitation programme. Progression to full activities was individualized among athletes. Return to normal activities and regular competition was allowed ~6 weeks after the intervention to enable the full immunomodulatory effect of ASCs. If necessary, patients were allowed to use acetaminophen, but the use of nonsteroidal anti‐inflammatory medication was not allowed. Complications and adverse events were recorded during follow‐up, if any. During competition, players underwent physical therapy in addition to their regular training.

### Statistical analysis

Data were processed with Stata software (StataCorp.). The normality of the distribution of numerical variables was verified using the Wilk–Shapiro test. Values are presented as the mean ± standard deviation (SD) or median and range for numerical variables and percentages for categorical variables. Paired *t*‐tests were used to compare score values at 6 months and 12 months to the baseline. The significance level of the statistical tests was set at *p* < 0.05.

## RESULTS

**Figure 1:**
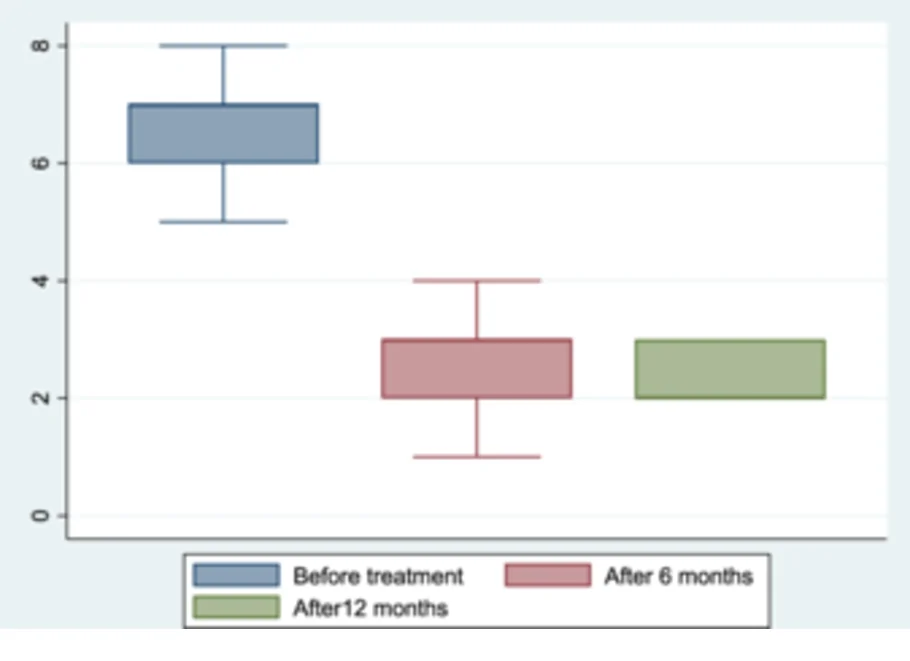
Numeric Pain Rating Scale (NPRS) before and after combined intra‐articular and intraosseous expanded adipose‐derived stromal cell knee injection in eighteen athlete's knees.

A total of 16 athletes (9 males and 7 females), 12 of them professional, aged 30.5 ± 2.8 years, with a body mass index of 24.8 ± 2.1 kg/m², preinjury Tegner scale 8.17 ± 1.38, participated in the study. None were excluded or lost in follow‐up. Two male participants presented bilateral knee involvement, treated simultaneously, resulting in a total of 18 knees treated. Among them, four knees (22.3%) had K‐L grade II OA, nine knees (50%) had grade III, and five knees (27.7%) had grade IV (all five had undergone previous partial meniscectomies) (Figure 1). Five athletes played handball, three played volleyball, three played soccer, two played basketball, one played tennis and two were runners.

Seventeen knees received both an IA plus IO injections targeting the femur and tibia, and one IA plus IO injections targeting the femur, tibia and patella. The injection sites were distributed as follows: six knees received medial injections, six received lateral injections and six received combined medial and lateral injections.

### Cell characterisation analysis

Following the International Society for Cellular Therapy (ISCT) recommendations (6), ≥95% of the MSCs population expressed CD105, CD73 and CD90, as measured by flow cytometry. Additionally, these cells lacked expression (≤2% positive) of CD45, CD34, CD14, or CD11b, CD79a, or CD19, and HLA‐DR (class II.20). The cells were able to differentiate to osteoblasts, adipocytes, and chondroblasts under standard in vitro differentiating conditions [8].

### Clinical scores

**Figure 2:**
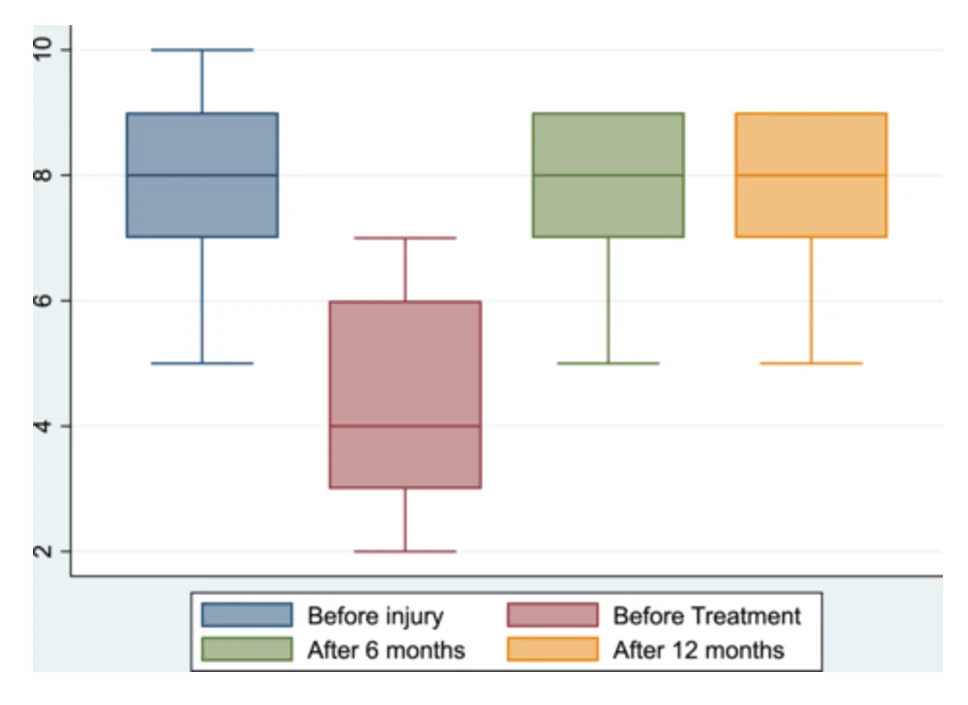
Box plot displaying Tegner scale before and after combined intra‐articular and intraosseous expanded adipose‐derived stromal cell knee injection in 18 athlete's knees.

**Figure 3:**
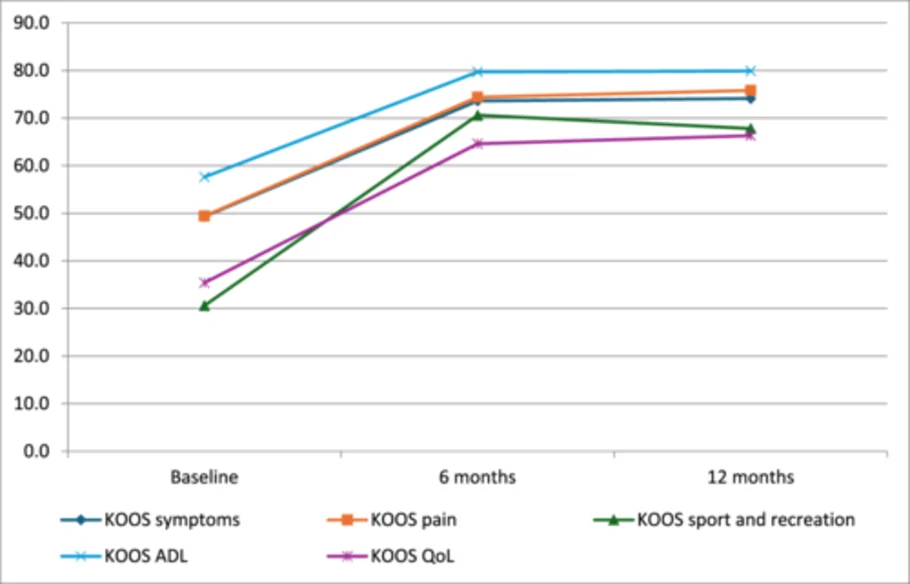
Mean values of KOOS subscales before and after combined intra‐articular and intraosseous expanded adipose‐derived stromal cell knee injection in 18 athlete's knees. ADL, Daily Leaving activities; KOOS, Knee Injury and Osteoarthritic Outcome Score; QoL, quality of life.

**Figure 4:**
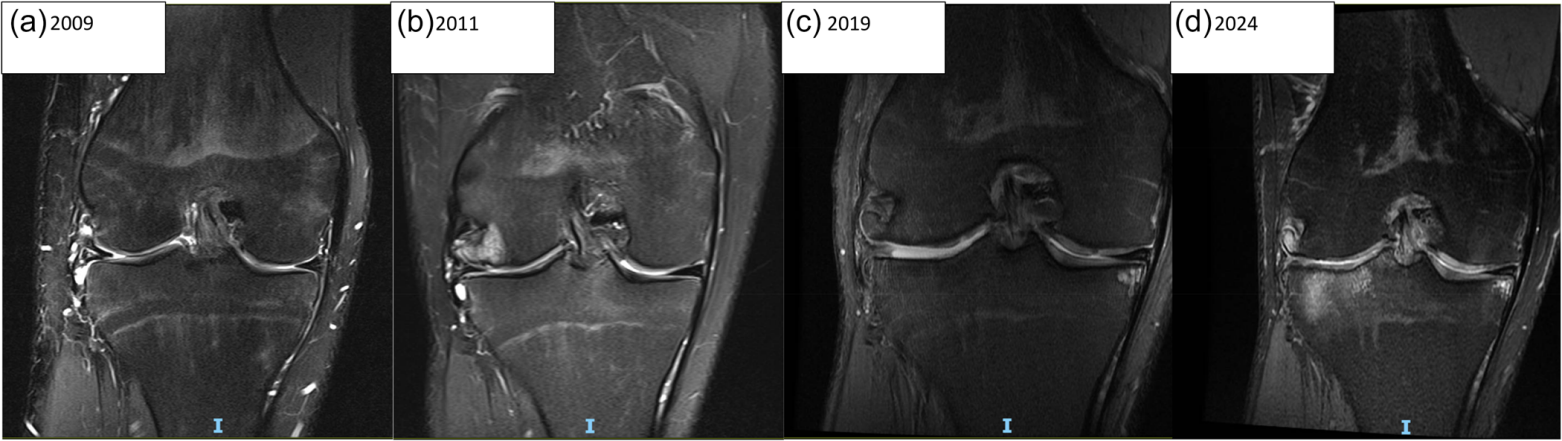
MRI Sequence of right knee injury in a professional handball player. (a) Lateral meniscal injury followed by meniscal repair. (b) Subchondral bone collapse after treatment. (c) Tricompartmental osteoarthritis. (d) Evolution of tricompartmental osteoarthritis 15 years later.

Patients showed significant improvement at 6 and 12 months compared to baseline in terms of NPRS, Tegner, KOOS symptoms, KOOS pain, KOOS sport and recreation, KOOS ADL and KOOS QoL (*p* < 0.0001) (Table 1, Figures 2 and 3). However, no significant differences were observed between the 6‐ and 12‐month outcomes, except for the KOOS sport and recreation subscale, which showed a mild yet significant decline (Figure 4). MCID for KOOS subscales was achieved in at least 16 of 18 knees at 6 and 12 months (Table 2).

**Table 1:** Clinical scores before and after combined intra‐articular and intraosseous expanded adipose‐derived stromal cell knee injection in eighteen athletes' knees.

Scale	Pretreatment (baseline)	6 months	*p* Value (baseline vs. 6 months)	12 months	*p* Value (6 months vs. 12 months)
NPRS	6.6 ± 0.9	2.6 ± 0.7	<0.0001	2.4 ± 0.5	0.7266
Tegner	4.4 ± 1.8	7.7 ± 1.2	<0.0001	7.8 ± 1.1	0.6875
KOOS symptoms	49.3 ± 6.4	73.6 ± 5.9	<0.0001	74.1 ± 4.9	0.7031
KOOS pain	49.4 ± 4.3	74.4 ± 5.8	<0.0001	75.8 ± 3.2	0.8691
KOOS sport and recreation	30.6 ± 6.2	70.6 ± 4.5	<0.0001	67.8 ± 4.3	0.0195
KOOS ADL	57.6 ± 3.6	79.7 ± 5.3	<0.0001	79.9 ± 4.8	0.8887
KOOS QoL	35.4 ± 10.3	64.6 ± 9.6	<0.0001	66.3 ± 5.3	0.4102

*Note*: All data are expressed in mean ± standard deviation.

Abbreviations: ADL, activities of daily living; KOOS, Knee Injury and Osteoarthritic Outcome Score; NPRS, Numeric Pain Rating Scale; QoL, quality of life.

**Table 2:** Absolute and relative frequencies of Minimal Clinically Important Difference (MCID) in subscales of Knee Injury and Osteoarthritic Outcome Score (KOOS) after 6‐ and 12‐months of combined intra‐articular and intraosseous expanded adipose‐derived stromal cell injection in eighteen athletes' knees.

KOOS	6‐month MCID	Reached MCID at 6 months^a^	12‐month MCID	Reached MCID 12 months^a^
Pain improvement	9.3	17 (94.4%)	9.1	18 (100%)
Symptoms improvement	8.4	18 (100%)	8.2	18 (100%)
Sport and recreation	9	18 (100%)	9.2	18 (100%)
Activities of daily living (ADL)	12.5	17 (94.4%)	11.6	17 (94.4%)
Quality of life (QoL)	10.3	17 (94.4%)	10.3	16 (88.9%)

^a^ Results are presented as percentages of knees that reached MCID (smallest difference between two measurements deemed necessary for a patient to perceive beneficial clinical improvement).

### MRI

**Figure 5:**
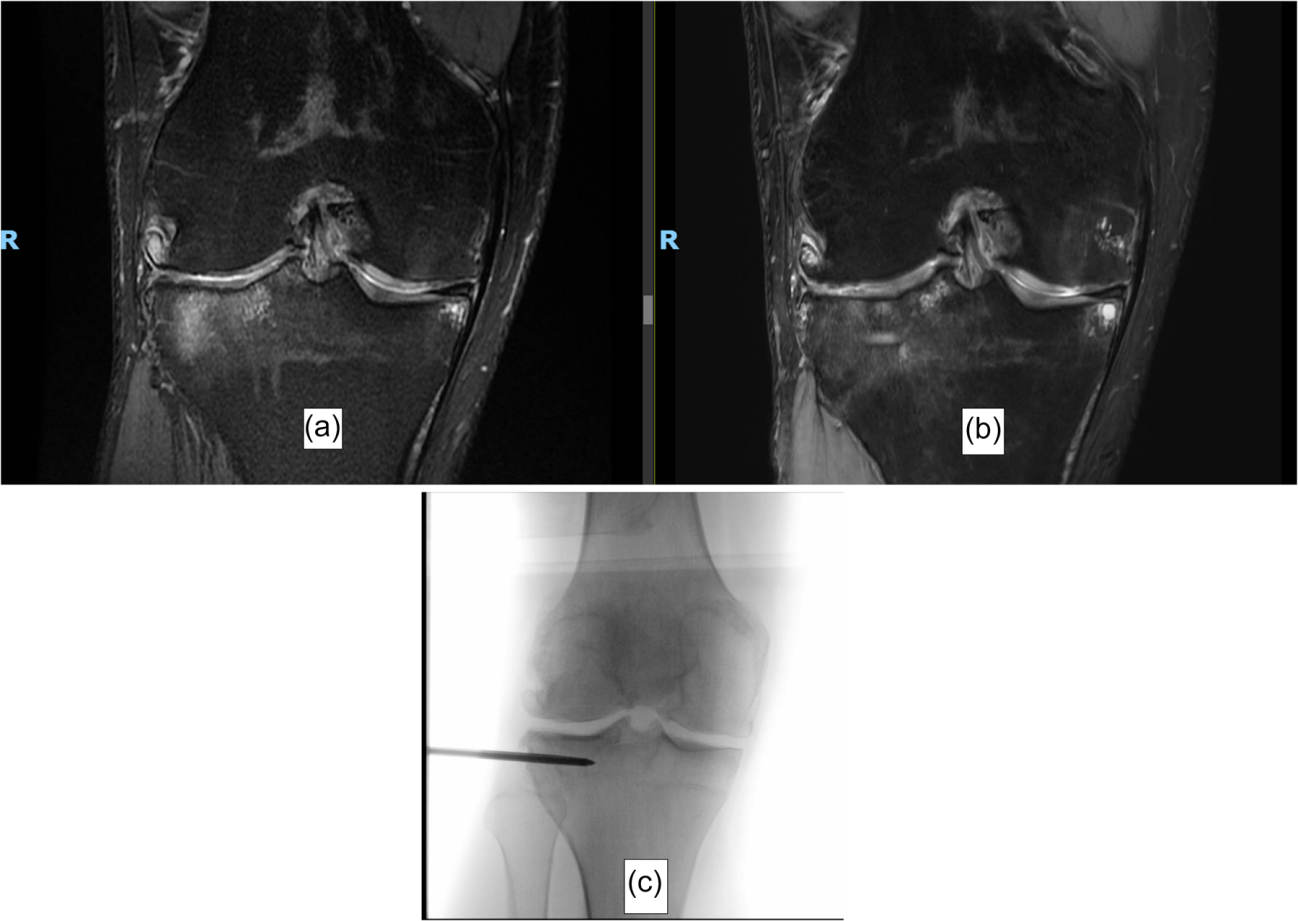
MRI coronal views. (a) Pretreatment, (b) 12 months postintraosseous MSCs injection, and (c) intraoperative procedure image in a 34 years old professional handball player. MRI, magnetic resonance imaging; MSCs, mesenchymal stromal cell.

MOAKS bone marrow assessment at 12 months revealed significant improvement in the following features: coronal femoral medial, coronal femoral lateral, sagittal femoral posterior, sagittal tibial posterior, patellar lateral, and cysts (Table 3, Figure 5). MOAKS articular cartilage was significantly improved in the coronal femoral medial, coronal femoral lateral, sagittal tibial medial, sagittal tibial posterior, and patella lateral features (Table 4).

**Table 3:** Magnetic Resonance Imaging Osteoarthritis Knee Score (MOAKS) bone marrow lesion evolution after 12 months combined intra‐articular and intraosseous expanded adipose‐derived stromal cell knee injection in eighteen athletes' knees.

MOAKS features	Bone marrow lesion (*n* = 18)			*p* Value
	Baseline median (range)	At 12 months median (range)	Number of knees improved	
Coronal femoral medial	2 (0–3)	1 (0–2)	8	0.0078
Coronal femoral lateral	2 (0–3)	1 (0–3)	6	0.0313
Coronal tibial medial	1 (0–2)	1 (0–2)	2	0.5000
Coronal tibial subspinous	0.5 (0–2)	0 (0–2)	3	0.2500
Coronal tibial lateral	1 (0–2)	1 (0–2)	4	0.1250
Sagittal femoral trochlea	1 (0–2)	1 (0–2)	4	0.1250
Sagittal femoral central	1 (0–2)	1 (0–2)	4	0.1250
Sagittal femoral posterior	2 (0–3)	1 (0–2)	7	0.0156
Sagittal tibial anterior	1 (0–2)	1 (0–2)	4	0.1250
Sagittal tibial middle	1 (0–2)	1 (0–2)	4	0.1250
Sagittal tibial posterior	1.5 (0–3)	1 (0–2)	7	0.0156
Patellar lateral	1.5 (0–3)	1 (0–2)	8	0.0391
Patellar medial	1 (1–3)	1 (1–2)	4	0.1250
Cysts	1 (0–2)	1 (0–2)	10	0.0020

**Table 4:** Magnetic Resonance Imaging Osteoarthritis Knee Score (MOAKS) articular cartilage pathology evolution after 12 months combined intra‐articular and intraosseous expanded adipose‐derived stromal cell knee injection in 18 athlete's knees.

MOAKS features	Articular cartilage pathology (*n* = 18)			*p* Value
	Baseline median (range)	At 12 months median (range)	Number of knees improved	
Coronal femoral medial	1 (0–3)	1 (0–2)	8	0.0078
Coronal femoral lateral	1.5 (0–3)	1 (0–2)	6	0.0313
Coronal tibial medial	1 (0–3)	1 (0–2)	3	0.2500
Coronal tibial subspinous	0 (0–1)	0 (0–1)	1	1.0000
Coronal tibial lateral	2 (0–3)	1 (0–2)	4	0.1250
Sagital femoral troclear	1 (1–2)	1 (0–2)	1	1.0000
Sagital femoral central	1.5 (1–3)	1 (1–3)	2	0.5000
Sagital femoral posterior	2 (0–3)	2 (0–3)	1	1.0000
Sagital tibial anterior	1 (0–2)	1 (0–2)	5	0.0625
Sagital tibial medial	1 (0–2)	1 (0–2)	6	0.0313
Sagital tibial posterior	2 (0–3)	1 (0–2)	6	0.0313
Patella lateral	2 (0–3)	1 (0–3)	6	0.0313
Patella medial	1 (1–3)	1 (1–2)	3	0.2500

### Complications and adverse events

Bruising was noted in all patients after the adipose tissue biopsy. A subcutaneous haematoma was observed in two patients, with further induration observed at the tissue biopsy harvest site. Although induration persisted for 2 months in one patient, both resolved without any treatment. All athletes experienced soreness at the site of the IO injection, and reported mild discomfort for 48 h after the IA injections. These adverse events were self‐limiting, requiring a period of unloading and analgesia, but not oral anti‐inflammatory medication. No severe adverse events were observed in any participants during the entire study period (up to 12 months postintervention).

## DISCUSSION

The main finding of this study was that athletes with symptomatic knee OA‐BMLs treated with a combination of IA and IO ASCs injections had effectively reduced pain, improved function, and improved MRI scores over a 12‐month follow‐up period, confirming our hypothesis. However, the KOOS sport and recreation subscale presented a mild yet significant decrease at 12‐month follow‐up compared to the 6‐month follow‐up, but remained higher than baseline and surpassed the MCID. This procedure also proved to be safe in the 18 treated knees.

BME on knee MRI examination is a common finding among athletes. Indeed, over 70% of collegiate basketball players show knee BME and cartilage injuries, but most of them are asymptomatic [30]. However, young athletes affected by OA‐BMLs have severe pain—a hallmark symptom—and inflammation. They are at a higher risk of rapid progression toward serious consequences, that is, end‐stage disease requiring arthroplasty due to cartilage and subchondral bone involvement [27, 40, 42].

IO injections in OA‐BMLs may play a role in helping patients improve after conservative treatment failure [2]. Moreover, active athletes are often too young for a knee replacement and are unlikely to undergo such a major surgery, which would likely threaten their sports career. IO PRP injections are gaining recognition; their use is simple, relatively inexpensive, and widely available, and they show a significant decrease in pain and increased functional scores in treating AO‐BMLs [6, 23].

The literature on the safety profile of ASCs in IO injections is limited [7]. Concerns about the ASCs' implantation within the bone and their impact on bone loss and diminished bone mineral density are similar to those associated with bone marrow adipocyte accumulation during ageing and its subsequent alteration of the microenvironment, affecting bone homoeostasis [1, 32]. However, ASCs' IA injections have proven safe, with no reported serious complications; however, mild and transient events, such as arthralgia and joint effusion, have been observed when injected intra‐articularly [17, 21, 33, 48]. In our series, no severe adverse events were observed in any participants during the study period.

Sanchez et al. [37], in a comparative study between IA PRP alone versus IA and IO PRP for severe knee OA (in 60 patients), revealed a significant improvement in all KOOS and WOMAC subscales (*p* < 0.05) up to a year. At the same time, the IA group did not improve in any of the scores. Moreover, sixteen out of 30 patients in the IA and IO group showed (i) minimal clinically important improvement compared to 8 out of 30 in the IA group at 6 months (*p* < 0.05), and (ii) 14 patients of the IA and IO group and 5 patients of the IA group at 1‐year follow‐up (*p* < 0.05). Similarly, Su et al. [41], in their randomised controlled trial, found that patients who received IA and IO PRP (administered twice, 2 weeks apart) had significant clinical outcomes, with sustained lower VAS and WOMAC scores and improvement in quality of life at 18 months follow up when compared to IA PRP and IA hyaluronic acid injection groups (*p* < 0.05, *n* = 82). These clinical outcome improvements are also accompanied by OA‐BMLs MRI imaging, as reported by Lychagin et al. [23], in which significant improvements in VAS, WOMAC, KOOS and Whole‐Organ Magnetic Resonance Imaging Score (WORMS) classification are observed up to one‐year follow‐up.

Furthermore, BMAC—an autologous product rich in progenitor cells and growth factors—has also been used to safely treat OA‐BMLs with promising results (in symptomatic patients but with no differences in MRI imaging using WORMS) [5, 7, 19, 23]. In light of existing evidence, the use of a combined IA and IO injection with ASCs has never been reported. This approach proved to be safe and showed promising clinical and radiological results. The treated athletes by this method reached a plateau of improvement around 6 months post‐intervention. Results were maintained at the 12‐month evaluation with similar values, except for Sport and Recreation KOOS, which presented a significant decrease. However, the latter scores were still significantly better than pre‐treatment values and surpassed the MCID. The decline in sport and recreation KOOS scores may be related to the highly demanding constraints imposed on the athletes in the present study.

The rapid improvement in the first 6 months after the procedure is likely due to the decompression effect of the treatment, the modification in training, and the absence of sports competition during the 6 weeks following the procedure. After this initial positive effect, sustained improvement up to the first year might result from the ASCs' injection and biological interplay with the knee joint, subchondral bone, and cartilage. Although there were previous studies related to IO injections, the present study′s results could not be directly compared to them due to the heterogeneity in terms of the study design, PROMs measured, and injection type used in patients from other publications, like PRP or BMAC [5, 13, 19, 23, 37, 41]. In fact, none of these studies involved the injection of expanded cells. All resulted in a significant decrease in pain and an increase in functional scores. Indeed, the present study, which included young active athletes, has comparable outcomes in that respect. In contrast, some studies performed subchondroplasty before the biological injection [5, 28], which prevents us from obtaining a reliable comparison. A previous study has shown that three monthly ASCs IA injections in OA knees outperformed leukocyte‐rich PRP [16]. Improvement was not only evident in cartilage evaluations but also in bone marrow's MOAK scores. We speculate that IO delivery of expanded MSCs allows them to dock at sites of BMLs in the subchondral bone, repopulating the damaged tissue and empowering resident cells, thereby shifting from a proinflammatory and profibrotic to a proregenerative cellular response [14, 29]. The latter healing process has probably led to an ordered deposition of extracellular matrix components. However, in vitro and in vivo studies are needed to confirm these hypotheses.

Although the mechanism of action by which IO injections are effective is not well known, our theory is that regular IA injections might not effectively reach the subchondral bone. Therefore, combined subchondral bone injections directly targeting the subchondral bone lesion result in a synergistic treatment with the IA injection effect on OA symptoms. In a rat model, PRP and BMAC IA and IO injections proved the most effective method for relieving joint pain [49]. The latter study has shown a rapid onset and long‐lasting effect on the subchondral bone. Moreover, we observed a decrease in pain‐associated mediators, such as calcitonin gene‐related peptide and substance P, expression. IA and IO injections provided greater protection against cartilage thickness loss, increased collagen type 2, and enhanced extracellular matrix (ECM) content compared to isolated IA injections [49]. These findings support previous studies highlighting the importance of targeting the subchondral bone microenvironment and structure in the progression of OA‐related pain and cartilage destruction [49].

The limitations of the present prospective study are related to the small number of treated knees, the absence of randomisation, and the lack of a control group. This precluded a significant subanalysis of the variables related to the outcome, including heterogeneous BMLs locations, and the role of the placebo effect in our results. NPRS and KOOS scores were obtained from patients, which are inherently subjective data. Additionally, we focused on sports‐related outcomes using the Tegner scale and KOOS sport and recreation subscale, but did not assess return to sports. Furthermore, an MRI evaluation was only performed at one point in time. Due to the short follow‐up period, it is impossible to determine whether this treatment alters the natural course of the disease. This initial study should be raised to a larger, longer follow‐up, high‐level, controlled study to confirm the efficacy of this treatment. Also, emphasizing the need for more accurate monitoring of the training load/regimen of the athletes postintervention.

The presented therapeutic option for OA‐BMLs treatment in athletes may represent an opportunity for them to alleviate pain with a minimally invasive procedure, with a short noncompetitive period of 6 weeks. The IA and IO injections of ASCs hold the potential to maintain athletic performance and career length while reducing disease progression.

In summary, combined IA and IO injections with ASCs are a promising and safe treatment option for OA‐BMLs in athletes and active patients. The procedure yielded clinical and radiological outcomes improvement for up to a year of follow‐up. Future studies are needed to test this combined treatment in a larger population of athletes and active patients with OA‐related BMLs.

## AUTHOR CONTRIBUTIONS

*Conceptualization*: Miguel A. Khoury. *Data curation*: Lorena Levi. *Formal analysis*: Theodorakys Marín Fermín, Lorena Levi. *Investigation*: All authors. *Data curation*: Theodorakys Marín Fermín, Lorena Levi. *Writing—original draft preparation*: All authors. *Writing—review and editing*: all authors. *Supervision*: Miguel A. Khoury, Karim Chamari. *Project administration*: Miguel A. Khoury, Theodorakys Marín Fermín, Montassar Tabben.

## CONFLICT OF INTEREST STATEMENT

The authors declare no conflict of interest.

## ETHICS STATEMENT

This prospective case series study was approved by the Ethical Committee of Regenerar® (Clinical Trial MR0024). Informed consent was obtained from all individual participants included in the study.

## Data Availability

The data underlying this article are available in the article and its online supplementary material.
